# A CDISC Implementation Maturity Model: Viewpoint on Moving Beyond Isolated Technical Fixes in Regulatory Submissions

**DOI:** 10.2196/105990

**Published:** 2026-11-13

**Authors:** Akash Kamble

**Affiliations:** 1 Independent Researcher, 4177 Gazebo St, Apt 6327, Aubrey, TX, 76227, United States, 1 4694425829

**Keywords:** Clinical Data Interchange Standards Consortium, CDISC, Study Data Tabulation Model, SDTM, Analysis Data Model, ADaM, implementation maturity model, clinical data standards, regulatory submissions, controlled terminology, Define.xml, metadata-driven transformation, Food and Drug Administration, FDA submission

## Abstract

The Clinical Data Interchange Standards Consortium (CDISC) has become the cornerstone of regulatory data submission frameworks for pharmaceutical and biotechnology organizations worldwide. Although the US Food and Drug Administration and Japan’s Pharmaceuticals and Medical Devices Agency require standardized study data, clinical data teams continue to face persistent operational barriers, including controlled terminology misuse, ambiguity in mapping protocol-specific data to domains, insufficient validation automation, and conformance findings that add review cycles. This Viewpoint argues that sustainable improvement in CDISC submission quality requires organizations to move beyond isolated technical fixes toward a staged, systematically assessable model of implementation maturity and proposes the CDISC Implementation Maturity Model (CIMM) as a framework for that assessment. Technical fixes—domain selection discipline, controlled terminology governance, template-based and metadata-driven automation, and multilayered validation—matter, but organizations that treat them as independent problems tend to underinvest in the institutional structures that make any one fix durable. Adapted from staged capability maturity modeling and established maturity model design procedures, the CIMM characterizes organizational CDISC capability across 5 dimensions—process standardization, automation coverage, terminology governance, validation integration, and institutional knowledge distribution—and 5 maturity levels, from Ad Hoc to Optimizing. Each of the 25 level-dimension cells carries an observable indicator, and an organization’s overall maturity is set by its weakest dimension. The framework is offered as a conceptual synthesis; its level definitions and the submission outcomes associated with each level are hypotheses to be tested, not validated findings. Submissions that are efficient to produce and more likely to clear regulatory review on the first attempt are more plausibly reached through the deliberate, staged development of the 5 capability dimensions the CIMM describes than through any single tool or technique. Testing the reliability of CIMM level assignment and its association with observed submission outcomes is the necessary next step.

## Introduction

The Clinical Data Interchange Standards Consortium (CDISC) has grown into the defining framework for organizing clinical trial data, providing a consistent structural and terminological foundation across pharmaceutical development programs worldwide. Both the Food and Drug Administration (FDA) and the Pharmaceuticals and Medical Devices Agency (PMDA) now require standardized study data in regulatory applications. In the United States, the requirement applies to studies starting after December 17, 2016, for new drug applications, abbreviated new drug applications, and biologics license applications, and studies starting after December 17, 2017, for commercial investigational new drug applications [1,2]. The FDA Data Standards Catalog lists which standard versions are supported and required at a given time, and the Study Data Technical Conformance Guide describes how they are to be applied [2,3]. Preparing standard-compliant submission components demands considerable knowledge and experience [4], and working knowledge of CDISC is now a core professional requirement for clinical data teams rather than optional background expertise.

Within the CDISC ecosystem, responsibilities are distributed across several interdependent components. Study Data Tabulation Model (SDTM) defines how tabulated data are organized for submission, Analysis Data Model (ADaM) governs the structure of datasets prepared for statistical evaluation, and CDASH (Clinical Data Acquisition Standards Harmonization) sets expectations for data capture at the point of collection through case report forms (CRFs). Alongside these are Define.xml, the Analysis Data Reviewer’s Guide (ADRG), and the Study Data Reviewer’s Guide (SDRG), collectively forming the metadata backbone that allows regulators to orient themselves within a submission, trace variable origins, and independently verify dataset contents [3,4].

Despite this well-defined architecture, many clinical organizations find that translating standards into submission-ready datasets is considerably more difficult in practice than it appears on paper. Recurring friction points include misapplication of controlled terminology, the difficulty of fitting protocol-specific instruments into SDTM domain structures not designed with them in mind, and the manual burden that falls on teams lacking mature automation infrastructure [5]. One academic research organization has described how its practice moved, across 3 successive trials, from converting data to SDTM after database lock to CDASH-based collection with ongoing conformance checking [5]. Accounts of this kind show that the transition is possible, but in the literature reviewed for this Viewpoint, the pathway from ad hoc practice to systematic, scalable implementation has received little structured attention.

This Viewpoint’s central position is that the recurring technical problems documented in the literature—terminology misuse, domain mapping ambiguity, and validation gaps—are symptoms of an underlying capability gap rather than independent defects to be patched one at a time. Organizations that fix a terminology problem without building terminology governance infrastructure, or that adopt a validation tool without integrating it into the development workflow, tend to see the same category of deficiency recur in the next trial. Maturity models have long been used to characterize such capability in software and information systems management [6,7], but none of those identified for this Viewpoint addresses the operational specifics of clinical data standardization for regulatory submission. This Viewpoint synthesizes existing implementation guidance and proposes the CDISC Implementation Maturity Model (CIMM), a 5-level conceptual framework intended to help fill that gap.

This Viewpoint draws on a narrative synthesis of published literature, regulatory and standards-body guidance, and documented practitioner experience relating to CDISC implementation for regulatory submission. It is not a systematic review: sources were selected purposively rather than through a reproducible search protocol, and backward citation tracking from included sources was used to identify further relevant work. The source base comprises peer-reviewed journals (the *Journal of the Society for Clinical Data Management*, the *Journal of Biomedical Informatics*, *JMIR Medical Informatics*, and *Clinical and Translational Science*), conference proceedings (PharmaSUG and PHUSE), standards-body publications (the SDTM and ADaM Implementation Guides and CDISC Controlled Terminology), and FDA and PMDA technical and guidance documents. The cited implementation literature, standards, and agency documents were published between 2014 and 2025, and web-based resources were accessed in September 2026; methodological works on maturity model development and on narrative review reporting, published between 1993 and 2019, were included regardless of date. Sources were prioritized when they documented implementation outcomes or represented governing standards and agency guidance. During revision, each cited source was reread against the statement it supports; sources that did not support their statements were removed or replaced, and each quantitative statement in this Viewpoint identifies the design and setting of its source.

The reporting of this synthesis was informed by the SANRA (Scale for the Assessment of Narrative Review Articles) criteria for narrative review articles [8]: the importance of the topic and the aims are stated in the *Introduction* section, the sources and their selection are described here, key statements are referenced, and the level of evidence behind quantitative claims is stated where those claims are made.

The synthesis was organized around 4 implementation domains: domain selection and controlled terminology governance; automation architecture for SDTM and ADaM dataset creation; validation and quality-control frameworks; and organizational practices spanning early planning, reusable component libraries, and version-transition management. From each source, recurring operational barriers and documented mitigation strategies were extracted and consolidated into the guidance presented in the sections that follow.

The CIMM adapts the staged structure of the Capability Maturity Model [6], in which each level builds on the practices of the level below, to the operational and governance dimensions of clinical data standardization. Its development followed the phases described by de Bruin et al [9] and the procedure model of Becker et al [10]. Scope was defined as organizational capability for producing CDISC-conformant study data for FDA and PMDA submissions. Five dimensions were selected by grouping the barriers and mitigations extracted from the sources into areas that can be assessed independently of one another. For each dimension, level indicators were then derived by ordering the documented practices from least to most institutionalized, and each indicator was phrased so that it can be confirmed from a documentary artifact. Maturity models developed for health information systems [7] informed the choice of a dimension-by-level structure, although those models address hospital information system management rather than regulatory data standardization. The later phases of maturity model development—testing the reliability of level assignment, deployment in organizations, and validation against outcomes—have not yet been carried out and are described under the *Future Directions* section. The CIMM is therefore a conceptual framework intended for organizational self-assessment and hypothesis generation.

## Domain Selection and Controlled Terminology

Deciding which SDTM domain should hold a given type of clinical data is one of the more nuanced judgment calls that data programmers and managers face. The SDTM Implementation Guide (SDTMIG) covers a wide range of data categories—Demographics (DM), Adverse Events (AE), Concomitant Medications (CM), Vital Signs (VS), Laboratory Tests (LB), Electrocardiogram (EG), and others—yet many protocol-specific assessments, particularly novel efficacy instruments or specialized safety monitoring tools, resist obvious assignment to any single domain. Getting this right depends on genuine working familiarity with what each domain is designed to capture: its structural logic, its required variables, and the kinds of data it handles well versus poorly [11].

Several practical strategies guide domain selection. Reviewing precedents from similar trials in the same therapeutic area reveals how regulators have previously accepted certain assessment types. Consulting SDTMIG domain templates and controlled terminology verifies that required variables and permitted values align with what was collected. When collected data do not fit a standard domain, supplemental qualifiers (represented in SUPP-- datasets) and sponsor-defined custom domains deserve serious consideration. An industry analysis of SUPP-- datasets across 325 studies from 124 sponsors, conducted by a validation software provider and presented at a conference, found that the number of nonstandard variables per study ranged from 1 to 618, with a mean of 84 and a median of 65.5 [12]. The same analysis found that only 44% of those nonstandard variables followed the recommended practice of a variable name prefixed with the parent domain code [12], suggesting that nonstandard variable handling is both common and inconsistently governed.

The SDTMIG permits sponsor-defined custom domains, which must be based on one of the general observation classes; 2-character domain codes beginning with X, Y, or Z are reserved for this purpose, although their use is optional [11]. The emphasis should be on deliberate, well-documented decision-making rather than convenience. The most defensible domain mapping decisions are treated as cross-functional commitments made during protocol development, not post hoc assignments made under time pressure during dataset preparation.

CDISC Controlled Terminology (CT) underpins the semantic consistency that makes cross-trial data comparison possible, and it is updated through regular published releases [13]. In the author’s experience, several patterns of misuse recur: teams apply nonstandard values when suitable standard terms already exist, use the same concept inconsistently across data points, fail to migrate to updated CT releases, or create sponsor-specific terminology where standard terms would have served just as well. Building a reliable governance structure requires dedicated infrastructure: terminology servers or reference datasets that feed current CDISC CT directly into transformation systems, validation rules that automatically flag nonstandard values, and cross-reference mappings between CRF-level terms and CDISC CT equivalents. Practitioner experience suggests that organizations with a centralized terminology management function achieve more consistent cross-study terminology than those relying on ad hoc, team-level decisions, although this has not been tested empirically (Table 1).

**Table 1.** Study Data Tabulation Model (SDTM) domain selection framework for common clinical assessment categories (author’s synthesis based on the SDTM Implementation Guide [SDTMIG] [11]).

Assessment category	Standard SDTM domain	Extension mechanism when applicable
Physiological measurements (eg, blood pressure, heart rate)	Vital Signs (VS)	Supplemental qualifiers (SUPPVS) for nonstandard qualifiers
Laboratory and biomarker evaluations	Laboratory Tests (LB)	Sponsor-defined custom Findings-class domain only where LB cannot represent the data; SDRG justification
Cardiac conduction parameters	Electrocardiogram (EG)	Category and subcategory variables (EGCAT, EGSCAT) or supplemental qualifiers (SUPPEG); custom domain only for data outside the EG structure
Patient-reported outcome instruments	Questionnaires (QS)	Sponsor-defined CT^a^ for proprietary scales
Novel efficacy composite endpoints	None (no standard domain applies)	Sponsor-defined custom domain based on the Findings class (eg, a reserved code beginning with X, Y, or Z), or Findings About (FA) where the data qualify an event or intervention; SDRG^b^ justification

a CT: Controlled Terminology.

b SDRG: Study Data Reviewer’s Guide.

## Automation for SDTM and ADaM Creation

Writing custom programs for every new trial is neither scalable nor safe from a quality standpoint. Template-based programming addresses this by capturing proven transformation logic in modular, reusable programs that read raw data, apply standard domain structures and controlled terminology, compute derived variables, and output SDTM-compliant datasets with consistent attributes [14]. Templates must be governed through formal code review and version control, and unit-tested against datasets with preverified correct outputs. A recent practitioner guide describes modular SAS macro and R package frameworks of this kind and reports case examples of shorter dataset creation times and high code reuse [14]. Those case examples are anonymized and are not accompanied by study design or measurement details, so they are best read as practitioner experience rather than as a measured effect.

At a more advanced level, metadata-driven transformation shifts from programmatic logic to declarative specification: a metadata repository captures which source variables feed which SDTM fields, how raw values map to controlled terminology, and what derivations and quality checks apply, and a generic transformation engine reads that metadata to produce compliant datasets without trial-specific programming for the bulk of the work [14,15]. The CDISC Operational Data Model has itself been used as much as a study metadata standard as a data exchange format [16], which makes it a natural basis for such repositories. This approach creates a bridge to automated Define.xml generation: when datasets and their metadata documentation originate from a single source of truth, discrepancies between data and regulatory documentation become structurally difficult to introduce [15]. A hybrid model, where metadata drive standard cases and custom code handles exceptions, tends to work best, since metadata-driven systems can struggle with highly complex or unusual transformation scenarios.

A parallel development is the CDISC Open Rules Engine (CORE), an open-source project that publishes CDISC conformance rules in machine-executable form together with a reference implementation engine, so that other rule engines can be checked against the same results [17]. In November 2023, CDISC began a 3-year research collaboration with the FDA’s Center for Drug Evaluation and Research and Center for Biologics Evaluation and Research to incorporate FDA Business Rules into CORE [18]. For organizations building metadata-driven pipelines, CORE’s metadata-centric design makes it a natural complement to domain mapping and terminology governance infrastructure.

One of the most impactful, and underused, strategies involves shifting validation from a terminal submission-preparation activity to a continuous, development-integrated process. In the academic research organization described above, SDTM datasets for the third trial were rebuilt from data exports every 2 weeks and checked with Pinnacle 21 on each cycle, which allowed issues to be resolved soon after they occurred rather than at database lock [5]. That account is a single-center opinion paper without a comparison group, but its logic generalizes: errors found early cost less to correct. Continuous validation requires institutional commitment beyond tool access: a triage process distinguishing genuine errors from acceptable, justified deviations, and a running issues log from first data available through submission so that SDRG and ADRG narratives can be constructed from complete records rather than reconstructed under pressure.

Because template libraries, metadata repositories, and rule engines generate data that are submitted to regulators, they are themselves computerized systems within the scope of good clinical practice. ICH E6(R3) expects computerized systems used in clinical trials to be validated in proportion to their risk to participant safety and to the reliability of trial results, and it sets expectations for audit trails, access control, and data governance across the data life cycle [19]. For a CDISC pipeline, this implies 3 controls. The transformation and validation tools should undergo risk-based validation, with the depth of testing scaled to each function’s effect on submitted data. The metadata repository should sit under change control with an audit trail, so that every change to a mapping, derivation, or codelist is attributable, dated, and linked to the datasets it affected. Those audit trails should be reviewed periodically. The FDA’s computer software assurance guidance, although written for medical device production and quality system software, offers a practical model for scaling assurance activities to risk [20]. These controls are built into the Level 4 and Level 5 indicators for process standardization and automation coverage in the CIMM (Table 2).

**Table 2.** Comparison of Clinical Data Interchange Standards Consortium (CDISC) automation approaches (author’s synthesis based on [14-16]).

Automation approach	Primary benefit	Key limitation	Best-fit scenario
Manual trial-specific programming	Full control over transformation logic	High effort; inconsistent across trials	Single-trial or highly novel end points only
Template-based programming	Reusable modules reduce per-trial effort	Requires upfront library investment	Multitrial programs with recurring domain types
Metadata-driven transformation	Declarative specification accelerates delivery	Complex edge cases handled less elegantly	Enterprise programs with standardized data collection
Hybrid metadata and custom code	Balances consistency with flexibility	Governance overhead for dual-track maintenance	Large organizations with diverse therapeutic portfolios
Fully automated pipeline with validation	End-to-end efficiency	Significant infrastructure, testing, and tool validation requirements	Organizations with mature CDISC governance

## Validation, Quality Control, and Documentation

21Comprehensive validation programs systematically check for missing required variables, invalid variable attributes, domain key errors, controlled terminology deviations, and cross-domain inconsistencies such as adverse event records referencing participant identifiers absent from the demographics domain [3,21]. The FDA and the PMDA classify conformance findings differently. The FDA no longer assigns formal severity to most of its validator rules; apart from a small set of rules tied to technical rejection, the FDA expects every unresolved issue to be explained in the reviewer’s guide [3,21,22]. The PMDA’s validation rules retain 3 severities: Reject, which suspends review until the issue is corrected; Error, which suspends review if the issue is neither corrected nor explained in advance; and Warning [22,23]. Commercial validators may add their own labels; Pinnacle 21 Enterprise, for example, types findings as error, warning, or notice [24]. These agency and vendor classifications describe the urgency of resolution. They are separate from the 3 validation layers proposed below.

This Viewpoint proposes that no single validation pass is sufficient and that validation be organized in 3 layers (Table 3). Technical validation establishes structural conformance; operational validation confirms internal data consistency and completeness; clinical validation verifies that datasets faithfully preserve clinical meaning and support protocol-specified end points. Clinical validation, as used here, is a review of the transformed SDTM and ADaM datasets to confirm that mappings, derivations, and flags preserve the meaning of the collected data. It differs from routine medical and data review, which examines the collected data for plausibility and protocol compliance before transformation, and it is best performed jointly by the statistical programmer, the trial statistician, and a clinical scientist or medical reviewer.

**Table 3.** Proposed multilayer Clinical Data Interchange Standards Consortium (CDISC) validation framework (author’s synthesis; agency rules as described in [3,23]).

Validation layer	Scope of checks	Regulatory objective	Typically performed by
Technical validation	Variable names, labels, types, lengths, structure	Confirms CDISC structural conformance	Statistical programmer, using a rule engine
Operational validation	Data completeness, referential integrity	Verifies internal consistency of submitted data	Statistical programmer and data manager
Clinical validation	Preservation of clinical meaning in transformed datasets; analysis alignment	Ensures datasets support protocol end points	Programmer, trial statistician, and clinical scientist or medical reviewer jointly
Define.xml consistency check	Alignment between metadata file and dataset contents	Supports regulatory reviewer navigation	Statistical programmer
Controlled terminology audit	Verification of CT^a^ values across variables	Ensures standardized terminology for cross-trial review	Terminology steward or data standards lead

a CT: Controlled Terminology.

The Define.xml file is the navigational backbone of the submission package, covering variable-level attributes, value-level metadata, dataset linkage information, and analysis results metadata for ADaM datasets [25]. The versions currently supported by the FDA are Define.xml v2.0 and v2.1, with v1.0 no longer supported for studies starting after March 2018 [2,25]; the PMDA publishes its own list of supported versions [23]. The most reliable prevention against Define.xml–dataset inconsistency is to generate it programmatically from the same metadata repository that drives SDTM and ADaM creation [15].

The SDRG and ADRG are too often treated as last-minute deliverables, but they serve an active quality-control function when maintained as living documents throughout trial conduct. A well-maintained SDRG forces the data team to articulate, in writing, the rationale for every nonstandard design decision—an articulation process that regularly surfaces latent inconsistencies before a reviewer encounters them.

## A Proposed CDISC Implementation Maturity Model

Organizational capability in CDISC implementation exists on a continuum rather than as a binary compliance state, yet the literature reviewed for this Viewpoint did not include a structured framework for characterizing where an organization sits on that continuum. This Viewpoint proposes the CIMM, a 5-level framework adapted from staged capability maturity modeling [6] to the operational and governance dimensions of clinical data standardization.

The CIMM characterizes organizational capability across 5 dimensions: process standardization (whether transformation and validation procedures are documented and consistently followed), automation coverage (the proportion of steps handled programmatically vs manually, and the governance of the tools that do so), terminology governance (whether CT management is infrastructure-supported and formally enforced), validation integration (how early in the trial life cycle conformance checking is applied), and institutional knowledge distribution (whether CDISC expertise is distributed across the team or concentrated in individuals). Table 4 gives an observable indicator for each dimension at each level.

A dimension is rated at a given level when the indicator for that level, and for every level below it, is consistently satisfied. Consistently satisfied means that the indicator is evidenced by documentary artifacts—for example, approved procedures, repository change logs, validation reports, issues logs, or training records—in every active study and in each of the organization’s 3 most recent submission packages, or in all of them if fewer than 3 exist. An organization’s overall maturity level is the lowest level achieved across the 5 dimensions, reflecting the view that a weak link in any single dimension constrains submission quality regardless of strength elsewhere.

**Table 4.** CDISC Implementation Maturity Model (CIMM) assessment matrix: observable indicators by dimension and level.

Dimension	Level 1: Ad Hoc	Level 2: Repeatable	Level 3: Defined	Level 4: Managed	Level 5: Optimizing
Process standardization	No written procedures; each study team sets its own approach	Informal checklists or work instructions exist; adherence is not verified	Approved procedures for SDTM^a^ and ADaM^b^ creation, validation, and Define.xml; deviations documented	Process metrics (eg, conformance findings per study, cycle times) defined and reviewed at set intervals	Procedures revised on the basis of portfolio-level trend analysis at a defined review cycle
Automation coverage	Study-specific programs written from scratch	Code copied from prior studies; no version control or unit tests	Version-controlled template library with code review and unit tests against reference outputs	Metadata repository drives most dataset creation and Define.xml generation; repository under change control with an audit trail	Risk-based validation evidence maintained for all pipeline tools; audit trails reviewed periodically; roadmap for emerging standards
Terminology governance	CT^c^ values chosen by individual programmers; CT version not recorded	CRF^d^-to-CT mappings kept per study; CT upgrades ad hoc	Central CT reference dataset or service; written upgrade policy; automated checks for nonstandard values	Impact assessment performed for each CT release; cross-study terminology consistency measured	New-term requests submitted to CDISC^e^; CT adoption planned ahead of releases
Validation integration	Validation performed only before submission	Validation at the end of dataset preparation; no structured issues log	Validation at predefined milestones; triaged issues log feeds SDRG^f^ and ADRG^g^	Scheduled automated runs from first data available; issue resolution time tracked	Validation outcomes trended across the portfolio and fed back into templates and standards
Knowledge distribution	CDISC decisions depend on one or two named individuals	Informal mentoring; knowledge held in shared folders	Role-based training curriculum; no single-person dependency for any critical function	Searchable cross-study library of mapping decisions and precedents	Staff take part in CDISC or PHUSE working groups; external learning fed into the internal library

a SDTM: Study Data Tabulation Model.

b ADaM: Analysis Data Model.

c CT: Controlled Terminology.

d CRF: case report form.

e CDISC: Clinical Data Interchange Standards Consortium.

f SDRG: Study Data Reviewer's Guide.

g ADRG: Analysis Data Reviewer's Guide.

Table 5 summarizes each level and states the submission outcomes that the model predicts. These outcomes are hypotheses for empirical testing, not demonstrated properties of organizations at each level. At Level 1 (Ad Hoc), implementation is trial-specific and undocumented, validation occurs only at submission preparation, and institutional knowledge is concentrated in a few individuals. At Level 2 (Repeatable), informal templates exist without formal governance, terminology mapping is case-by-case, and validation occurs at the end of dataset preparation without a structured issues log. At Level 3 (Defined), organizations have documented procedures for transformation, validation, and Define.xml creation, template libraries carry code-review requirements, CT is managed centrally, validation is integrated at defined milestones, and staff are cross-trained. At Level 4 (Managed), metadata-driven transformation under change control handles most dataset creation, Define.xml and reviewers’ guides are generated programmatically from a shared metadata repository, and quantitative quality metrics drive process improvement. At Level 5 (Optimizing), organizations maintain risk-based assurance for their tooling, improve continuously from portfolio-level performance data, contribute to CDISC standards development, and anticipate version transitions through structured impact assessments before new versions reach active submissions.

Organizations can apply the CIMM through structured self-assessment against the Table 4 indicators, identifying the capability gaps most likely to affect submission quality and prioritizing investment accordingly. Because each indicator is tied to a documentary artifact, 2 assessors rating the same organization should be able to reach the same level; whether they do in practice is an empirical question addressed under the *Future Directions* section. Unlike a 1-time compliance audit, the CIMM is designed for periodic reassessment, allowing organizations to track improvement and to test whether maturity advancement corresponds to measurable changes in submission quality metrics.

**Table 5.** CIMM level summaries and hypothesized submission outcomes.

Level	Level name	Core characteristics	Hypothesized outcomes (to be tested)
1	Ad Hoc	Trial-specific programming; no reuse; validation only before submission; knowledge concentrated in individuals	High and variable conformance finding counts; unpredictable cycle times
2	Repeatable	Informal template sharing; end-of-preparation validation; limited cross-study consistency	Fewer findings than Level 1, with wide variation between studies
3	Defined	Approved procedures; governed template library; centralized CT^a^; milestone-based validation	Consistent finding counts and predictable cycle times across studies
4	Managed	Metadata-driven transformation under change control; programmatic Define.xml, SDRG^b^, and ADRG^c^; continuous validation; tracked metrics	Low first-cycle conformance findings; short database-lock-to-submission times
5	Optimizing	Portfolio trend analysis fed back into standards; risk-based tool assurance; CDISC^d^ or PHUSE working group participation	Lowest and most stable first-cycle findings in the portfolio

a CT: Controlled Terminology.

b SDRG: Study Data Reviewer's Guide.

c ADRG: Analysis Data Reviewer's Guide.

d CDISC: Clinical Data Interchange Standards Consortium.

## Complex Scenarios and Organizational Practice

Even with thorough SDTMIG familiarity, most trials collect data resisting clean domain assignment. The SDTMIG provides extension mechanisms: sponsor-defined custom domains, SUPP-- datasets for nonstandard variables alongside standard domain records, grouping variables, and sponsor-defined controlled terminology [11]. The industry analysis summarized above, with up to 618 nonstandard variables in a single study [12], underscores how central nonstandard variable handling is to routine SDTM implementation. Thorough SDRG documentation of the rationale for custom elements transforms what might otherwise appear as a compliance gap into a transparent, defensible design decision.

The Basic Data Structure (BDS) within ADaM handles most continuous and categorical end points effectively, but responder definitions, time-to-event evaluations with complex censoring, composite end points, and irregular repeated measures require careful structural design before any code is written [26,27]. Derived flags and grouping variables created consistently across trials facilitate integrated summaries; traceability from ADaM back to underlying SDTM data gives reviewers the ability to independently verify how evaluation variables were derived [26].

Perhaps the single most impactful organizational change is moving CDISC planning upstream into protocol development. In the academic research organization’s account, converting a 2017 trial whose case report forms had not been designed with CDISC in mind took an estimated 1120 hours, about 7 months of one full-time equivalent, whereas a 2018 trial built on CDASH-compliant forms took about 320 hours; free-text fields that could not be mapped to standard variables ended up in the Comments domain [5]. These are retrospective estimates from a single organization, and the second conversion was outsourced, so they indicate direction rather than effect size. Therapeutic area user guides show the same principle at the level of a disease area: shared data structures and terminology allow data from separate studies to be integrated [28]. For organizations running multiple trials, a well-maintained, version-controlled component library-transformation programs, terminology mappings, ADaM templates, validation checks, and Define.xml structures—compresses timelines and reduces the surface area for error. CDISC version transitions require similar governance. The FDA Data Standards Catalog records the dates from which each standard version is supported and required [2], and the author recommends a defined adoption boundary for new trials, a structured impact assessment before transition, and parallel dataset builds comparing old- and new-version outputs before the new version is used for a real submission. Changing versions mid-trial should be treated as a last resort given the reprocessing and revalidation burden involved.

## Discussion

### Principal Findings

The strategies synthesized above—domain selection discipline, terminology governance, automation architecture and its governance, layered validation, and organizational practice—reflect published implementation experience and agency guidance across teams at varying stages of CDISC maturity. The central position of this Viewpoint is that these strategies are not independently sufficient: an organization that adopts one without the institutional structure to sustain it tends to see the same category of deficiency recur. The CIMM’s contribution is a structured vocabulary and an assessable set of indicators for characterizing organizational capability, together with a set of testable predictions about how capability relates to submission outcomes.

The automation and validation strategies described here are converging with a broader shift toward machine-readable submission formats. CDISC developed Dataset-JSON as a successor to the SAS version 5 transport (XPT) format [29]. Following a pilot with CDISC and PHUSE, the FDA published a notice in 2025 requesting comments on accepting Dataset-JSON version 1.1, which it described as having the long-term potential to replace XPT [30]; sponsors should confirm the exchange formats accepted at the time of submission in the current Data Standards Catalog [2]. For organizations already operating metadata-driven pipelines, this transition is comparatively low-friction, since the same metadata repository driving SDTM and ADaM creation can generate Dataset-JSON output alongside XPT as regulatory acceptance expands. CDISC 360i, launched in 2025, extends this principle further, aiming to turn standards into executable metadata that link digital protocols and biomedical concepts through to dataset specifications and analysis results [31]. Organizations at CIMM Level 4 and above are likely to be best positioned to adopt these initiatives, since the metadata-centric infrastructure such organizations already maintain is the same infrastructure these formats assume.

### Limitations

The CIMM is a conceptual framework derived from synthesis of published implementation literature rather than empirical validation against a representative sample of clinical data organizations. Level boundaries and indicators reflect the author’s interpretation of documented best practices; they have not been tested through direct organizational assessment, nor has interrater reliability for level assignment been established. The sources underpinning the CIMM indicators are predominantly conference proceedings and practitioner accounts reflecting large-sponsor, FDA- and PMDA-oriented practice, which may bias the level definitions toward resource-intensive practices. Because sources were selected purposively rather than through a reproducible search, relevant work may have been missed, including any existing maturity framework specific to clinical data standardization. Several of the quantitative figures cited come from a single organization or from a vendor analysis, and none comes from a controlled comparison. The strategies described reflect practices primarily documented for FDA and PMDA submissions; applicability to real-world evidence studies, postapproval programs, or other regulatory contexts may differ. This Viewpoint also does not account for the considerable variation in feasibility and cost across organizations of different sizes: the economic case for metadata-driven transformation differs substantially between large pharmaceutical companies and small to mid-size biotechnology sponsors.

### Future Directions

Empirical validation of the CIMM should proceed in 2 steps. First, the reliability of level assignment should be tested by having independent assessors rate the same organizations against the Table 4 indicators. Second, assessed levels should be correlated with observable submission outcomes, such as first-cycle conformance findings, agency information requests on study data, and database-lock-to-submission times, in a representative organizational sample. Investigation of the cost-effectiveness of specific automation investments at each maturity level, stratified by organization size, would provide economic evidence for resource decisions. As adoption of CDISC CORE, Dataset-JSON, and CDISC 360i matures, new implementation challenges specific to machine-executable rule sets will likely emerge, and the use of CDISC standards for real-world data raises its own barriers, including mapping from health care data models [32], that current frameworks do not yet anticipate.

### Conclusions

For clinical data organizations, submissions that are efficient to produce and more likely to clear regulatory review on the first attempt are more plausibly reached through the deliberate, staged development of process standardization, automation coverage, terminology governance, validation integration, and distributed institutional knowledge than through any single tool or technique. The CIMM proposed here offers a starting vocabulary and an assessable set of indicators for that development; testing its reliability and its association with observed submission outcomes is the necessary next step.

## Abbreviations

ADaM: Analysis Data Model
ADRG: Analysis Data Reviewer's Guide
BDS: Basic Data Structure
CDASH: Clinical Data Acquisition Standards Harmonization
CDISC: Clinical Data Interchange Standards Consortium
CIMM: CDISC Implementation Maturity Model
CORE: CDISC Open Rules Engine
CRF: case report form
CT: Controlled Terminology
FDA: Food and Drug Administration
PMDA: Pharmaceuticals and Medical Devices Agency
SANRA: Scale for the Assessment of Narrative Review Articles
SDRG: Study Data Reviewer's Guide
SDTM: Study Data Tabulation Model
SDTMIG: SDTM Implementation Guide
